# Fertility Counseling in Survivors of Cancer in Childhood and Adolescence: Time for a Reappraisal?

**DOI:** 10.3390/cancers13225626

**Published:** 2021-11-10

**Authors:** Francesca Filippi, Fedro Peccatori, Siranoush Manoukian, Carlo Alfredo Clerici, Chiara Dallagiovanna, Cristina Meazza, Marta Podda, Edgardo Somigliana, Filippo Spreafico, Maura Massimino, Monica Terenziani

**Affiliations:** 1 Infertility Unit, Fondazione IRCCS Ca’ Granda Ospedale Maggiore Policlinico, 1-20133 Milan, Italy; francesca.filippi@policlinico.mi.it (F.F.); chiara.dallagiovanna@policlinico.mi.it (C.D.); dadosomigliana@yahoo.it (E.S.); 2 Fertility and Procreation Unit, Division of Gynaecologic Oncology, IEO European Institute of Oncology IRCCS, 1-20133 Milan, Italy; fedro.peccatori@ieo.it; 3 Medical Genetics Unit, Fondazione IRCCS Istituto Nazionale dei Tumori, 1-20133 Milan, Italy; siranoush.manoukian@istitutotumori.mi.it; 4 Department of Oncology and Hemato-Oncology, Università degli Studi di Milano, 1-20133 Milan, Italy; carloalfredo.clerici@istitutotumori.mi.it; 5 Pediatric Oncology Unit, Fondazione IRCCS Istituto Nazionale dei Tumori, 1-20133 Milan, Italy; cristina.meazza@istitutotumori.mi.it (C.M.); marta.podda@istitutotumori.mi.it (M.P.); filippo.spreafico@istitutotumori.mi.it (F.S.); maura.massimino@istitutotumori.mi.it (M.M.); 6 Department of Clinical Sciences and Community Health, Università degli Studi di Milano, 1-20133 Milan, Italy

**Keywords:** hereditary cancer syndrome, cancer survivors, fertility preservation, genetics, infertility, preimplantation genetic testing, oocyte donation, adoption

## Abstract

**Simple Summary:**

Genetic predisposition to a disease obliges women to face the fear of transmitting cancer to their offspring. This might affect their willingness to seek pregnancy and adhere to fertility preservation programs. This often-neglected issue should be discussed during fertility counseling, and patients should be offered options to overcome the problem (i.e., PGT-M, egg donation and adoption). This opinion paper arose from the authors’ multiple discussions and meetings on this subject.

**Abstract:**

Genetic predisposition could have an important role in the pathogenesis of cancers in children and adolescents. A recent study by our group showed that, among female survivors of cancers in childhood and adolescence, the proportion of cases involving a possible genetic predisposition was sizable (at least one in five). Our sample is too small to be representative of the general population, but it gave us an opportunity to reappraise this issue. Women with a genetic predisposition can transmit the risk of cancer to their offspring, and their awareness of this may influence their reproductive and fertility preservation choices. In our experience, a predisposition to cancer receives little attention in the fertility counseling and decision-making process unless a patient already has a definitive molecular diagnosis of a hereditary cancer syndrome. We feel it is essential to empower women on this issue, particularly as there are ways to overcome the problem, including preimplantation genetic testing (PGT-M) in definitively diagnosed cases, egg donation and adoption. In the context of fertility counseling for survivors of cancer in childhood and adolescence who have reached adulthood, the risk of transmitting a predisposition to cancer should be discussed with patients, if relevant and desired.

## 1. Introduction

Female survivors of cancer in childhood and adolescence form a population with specific issues. Gynecological assessment and fertility counseling before and after these patients complete their cancer treatment is of the utmost importance [1,2,3,4]. There is a general consensus that pediatric and adolescent cancer patients should be offered fertility preservation at the time of their diagnosis [5,6] or, in selected cases, when they reach adulthood [5,7]. In a recent study, we retrospectively reported on all young women off therapy for childhood cancer who attended our Oncofertility Service over a 5-year period. From 2015 onwards, we systematically counseled young women with a history of cancer in childhood or adolescence. This service was free of charge. Information was obtained from medical records. Counseling was provided for 126 women, 36 of whom either did not complete the assessment or were too young, leaving a total of 90 subjects. We documented a decreased ovarian reserve and premature ovarian insufficiency in 35 (39%) and 19 (21%) cases, respectively. Overall, three in every five of these women in fact faced premature exhaustion of their ovarian reserve upon reaching adulthood. In total, 13 women with a reduced, but not exhausted, ovarian reserve were eligible for oocyte cryopreservation, and 9 of them (69%) underwent ovarian hyperstimulation to freeze their oocytes as part of a fertility preservation program [8].

## 2. Fertility Counseling in Childhood and Adolescent Cancer Survivors

Genetic predisposition could have an important role in the pathogenesis of cancers in children and adolescents [9,10,11], but little has been done to investigate how harboring a transmissible gene mutation might affect the desire for future parenthood and fertility preservation uptake. We therefore reassessed our series of 90 female survivors of cancer in childhood or adolescence [8], inferring the proportion likely to have a genetic predisposition to cancer. We identified 14 women who had two primary neoplasms, and 2 who had three, for a total of 16/90 (17%) that met the clinical criteria suggesting a genetic predisposition to cancer. Five women also had siblings with a history of cancer; one had a mother with the same neoplasm as her own; and a specific genetic disorder had already been documented in three cases (two patients with the APC pathogenic variant, and one with the RB1 pathogenic variant). Details of these 20 cases (22% of the cohort) are provided in Table 1. We thus inferred that the proportion of cases with a genetic predisposition in our sample could be sizable—at least one in five—and higher than is generally assumed and reported in the literature [12]. Despite the small size of our sample, which prevents us from generalizing from our results, we believe these findings deserve careful consideration.

About 80% of adolescent and young adult cancer survivors report feeling uncertain about their future family-building prospects, but, if questioned, having a biologically related child is generally cited as a “first choice” [13]. Evidence for the risk of cancer in the offspring of subjects who had a cancer during childhood or adolescence is scant and contrasting. It is noteworthy that, as highlighted in a recently published editorial, up to 18% of children diagnosed with cancer harbor a germline mutation that predisposes them to subsequent malignant neoplasms [14]. These data are consistent with our findings. In contrast, a study on cancer survivors’ offspring conducted by a German group on 1239 women found that their health status was comparable with that of the general population [15].

Alongside the discussion about the magnitude of the risk of cancer in the offspring of cancer survivors, it is worth noting that no studies have yet investigated the possible role of cancer survivors’ awareness of a genetic predisposition on their decisions regarding parenthood and fertility preservation [6]. It is of the utmost importance to bear in mind that a genetic predisposition may not always be identified by means of molecular analyses, and women may remain at risk of vertical transmission even in the absence of a definite diagnosis. Many factors—including ethical, religious, cultural and socio-economic variables, and the information individuals receive—can influence whether they choose to undergo fertility preservation. A suspected or known genetic predisposition to cancer, and the risk of transmitting this condition to one’s offspring, may be one such factor. A greater awareness of this aspect could arguably influence any decision to undergo fertility preservation or try to become pregnant, be it naturally or with the aid of the previously stored material. Our experience indirectly confirmed that the possible transmission of a predisposition to cancer was not perceived as a concern by our population. Demand for our fertility counseling service was high, and women’s choices (regarding storing oocytes or anticipating efforts to become pregnant) did not reflect any worries about the risk of transmitting a predisposition to cancer to their offspring, nor did the women themselves actively ask for clarification on this issue [8].

The topic of motherhood for young cancer survivors is multifaceted and, in our opinion, should not be limited to discussing technical and procedural issues. The risk of transmitting their disease to their children could be an important concern for any woman with a history of cancer in childhood and adolescence. This worry could be amplified if a hereditary cancer syndrome or multiple cancers have been diagnosed in the same person or their siblings. This is an issue with complex psychological and ethical implications. From a psychological perspective, individuals having to make decisions about their reproductive options may be vulnerable and exposed to the detrimental effects of receiving incomplete or biased information. Patients’ decisions should not be based on their feelings alone. They should be oriented by an objective, in-depth knowledge of the issues involved and expressed in the context of a supportive, thorough and multidisciplinary counseling process that provides an adequate space for the emotional, affective and relational aspects to emerge.

A considerable number of the young women in our sample had a personal and/or family history pointing to a genetic predisposition (Table 1), which is a factor to consider when cancer develops in childhood or adolescence [11]. Whether and to what degree being aware of such a genetic predisposition to cancer interferes with an individual’s subsequent family planning remains to be examined. When clinically indicated, comprehensive genetic counseling and testing should be offered, ensuring that all necessary support is subsequently available [16]. This could be important for cases at high risk, particularly as there are options that enable the problem to be overcome, including preimplantation genetic testing (if a definite diagnosis has been established), egg donation or adoption.

Pre-implantation genetic testing for monogenic disorders (PGT-M) might be an option to avoid those with a definite molecular diagnosis giving birth to offspring at high risk of inherited cancer. This approach has been considered ethically acceptable by the Ethics Committee of the American Society for Reproductive Medicine [17], but it has some important limitations. First, only patients with highly penetrant monosomic gene mutations could benefit from such a procedure (as was the case for three women in our cohort). Second, as most known causes of genetic predisposition have a dominant inheritance, the success rate can be expected to be halved, and this issue could be particularly important in the case of multigenic conditions. It is also important to bear in mind that, in most cases, any such tests have to be performed on previously stored material and, therefore, on a limited number of oocytes. Third, the costs of such procedures are significant and are rarely covered by public health systems, so there is a considerable risk of this approach exacerbating inequalities.

In the field of assisted reproduction, fertility preservation and PGT-M are not the only potentially useful methods that could be offered to female survivors of cancer in childhood and adolescence who wish to have children. Egg donation is a further viable option. This clinical option has become socially acceptable, and its use has been spreading in recent years [18], with public health coverage assured in many countries. Egg donation is unquestionably indicated for women with an exhausted ovarian reserve who did not undergo fertility preservation and for those who did but then failed to produce offspring with their own stored material. It would also be reasonable to say that another indication for egg donation may be female cancer survivors with a genetic predisposition to cancer in order to avoid the risk of transmitting this condition to their children. We do not discourage efforts to have their own biological child, but we feel the need to inform women at high risk of carrying a genetic predisposition about all available options so that they can make an informed choice. Egg donation is already frequently considered for women with exhausted ovarian reserves who did not undergo fertility preservation at the time of their cancer diagnosis or who did but were still unable to have children. There are issues with cultural beliefs that can be overcome for some, but not all, women. The complexity of egg donation also hampers thorough counseling at the time of a cancer being diagnosed, when patients and their relatives may be overwhelmed by the gravity of the diagnosis, and making a well-considered decision about future parenthood could be challenging [19]. There is also the fact that people may change their mind about parenthood over time, and this is particularly true of younger people.

According to the literature, about 60% of pediatric and adolescent cancer patients are not informed about assisted reproductive technologies. More than 80% would reportedly consider adoption, but 88% of these patients express concern about the process involved [20]. Medical, psychological, ethical and legal issues are all possible obstacles to adoption. The “White Paper on the needs of young people living with cancer” [21] issued by Youth Cancer Europe refers to the “right to be forgotten”, but guidelines on how a previous pediatric neoplasm affects the risk of being refused the chance to adopt a child have not been established, and this matter would demand an analytical effort and a consensus, starting with the necessary medical and legal background work.

The above-mentioned publication about our systematic fertility counseling [8] was an opportunity for reflection and self-criticism. Even if evidence emerging from our experience cannot be generalized because of the relatively small sample size and the study’s single-center design, some important issues should be emphasized. As professionals who work with fertility specialists and are involved in the care of children and adolescents with cancer we wrongly assumed that women at risk of transmitting a predisposition to disease would worry about the health of their future children, fear transmitting them a deleterious genetic condition or dying and leaving them motherless. This was not the case. The majority of the women in our sample of survivors of cancer in childhood and adolescence completed their proposed gynecological assessments (79%), most of the women offered oocyte cryopreservation agreed to the procedure (69%), and a fair proportion of the sample started to try and become pregnant naturally soon after receiving counseling (29%) [8]. The question “Will my child go through what I went through?” was not a barrier in our series. This finding is at odds with reports that women with the BRCA mutation express concern about how to deal with the possibility of their children having the mutation as well [22]. Reasons that may explain this inconsistency include the younger age at diagnosis of patients with cancer in childhood and adolescence, and the different types of cancer in our cohort. We simply assumed that concern about gene mutation transmissibility would discourage women from undertaking any fertility counseling and post-cancer fertility preservation, but we were mistaken. We wanted to avoid overwhelming our young patients with additional stressful issues at the time, but this may not have been the most effective approach. It should be noted, however, that the women in our sample were not asked explicitly about their feelings and fears concerning the risk of transmitting their disease, making it difficult for us to provide clear evidence on this matter.

In the context of fertility counseling for survivors of cancer in childhood and adolescence who reach adulthood, referral for genetic counseling is mandatory if women fulfill the specific criteria for testing and have not already been assessed [16]. This approach may be highly challenging and should be used wisely to avoid generating unjustified fears or prompting drastic decisions (to definitively abandon the idea of motherhood, for instance). Parallel psychological support is also warranted. Receiving fertility counseling could be emotionally challenging, and any considerations about the genetic risk of transmitting a susceptibility to cancer can make things worse. Reproductive choices in patients with a history of malignancy need to be supported by dedicated specialists to address any fears regarding cancer recurrences and the risk of leaving a child orphaned. Finally, it is worth emphasizing that a psychological support network is of paramount importance for cancer survivors who are diagnosed with insurmountable infertility. They may experience a sense of profound disappointment at the end of a prolonged period of emotional expectations and investments.

## 3. Conclusions

In conclusion, fertility counseling for adolescent and young adult cancer survivors is an emerging and challenging area of care. It provides an opportunity for discussing further, and in more depth, issues that were first addressed at the time of the cancer’s diagnosis, sometimes without involving the patient. Fertility counseling for survivors should be seen as the second phase of a process that began when their cancer was diagnosed. This second phase can entail a realistic and in-depth discussion of the individual’s reproductive situation and future prospects in a non-urgent and more mature setting. In women with syndromes predisposing to cancer, counseling should also incorporate a discussion about the risk of vertical transmission and options to prevent it.

## Figures and Tables

**Table 1:** Characteristics of the 20 women included.

Patient	1st Neoplasm	2nd Neoplasm	3rd Neoplasm	Family History/Genetic Variant
1	Metastatic neuroblastoma	Ossifying fibroma	Breast hamartoma	
2	Hodgkin lymphoma	Pulmonary synovial sarcoma		
3	Nasopharyngeal carcinoma	Adrenal ganglioneuroblastoma		
4	CNS ^a^ germinoma	Ovarian borderline tumor		
5	Wilms tumor	Uterine STUMP		
6	Ewing sarcoma	Osteogenic sarcoma		
7	Ewing sarcoma	Melanoma		
8	Non-Hodgkin Lymphoma	Ewing sarcoma		Mother with breast cancer (at 38 years old)
9	Ovarian germ cell tumor	Ovarian cystic adenoma		
10	ALL ^b^	Myoepithelioma		
11	Osteogenic sarcoma	Thyroid carcinoma		Sister with thyroid carcinoma (at 21 years old)
12	Ovarian mature teratoma	Ovarian mature teratoma		Mother with ovarian teratoma and thyroid carcinoma
13	Extraosseous Ewing sarcoma	Salivary gland adenocarcinoma	Plummer adenoma	Paternal grandmother with endometrial carcinoma (MSH6 variant of unknown significance of paternal origin)
14	Leptomeningeal sarcoma	Breast cancer		
15	Medulloblastoma	Melanoma		FAP ^c^ (APC pathogenic variant)
16	Medulloblastoma	Desmoid tumor		FAP ^c^ (APC pathogenic variant)
17	Ovarian germ cell tumor			Brother with CNS ^a^ low-grade glioma (at 4 years old)
18	Neuroblastoma			Sister with CNS ^a^ germinoma (at 7 years old)
19	Bilateral synchronous ovarian germ cell tumors			Sister with ovarian teratoma (at 22 years old)
20	Bilateral retinoblastoma			Brother with Hodgkin lymphoma (at 10 years old) (RB1 pathogenic variant)

^a^ CNS: central nervous system, ^b^ ALL: acute lymphoblastic leukemia, ^c^ FAP: familial adenomatous polyposis.
